# Energy Metabolism in Gynecological Cancers: A Scoping Review

**DOI:** 10.3390/ijerph19116419

**Published:** 2022-05-25

**Authors:** Ana Paula Pagano, Katherine L. Ford, Kathryn N. Porter Starr, Nicole Kiss, Helen Steed, Janice Y. Kung, Rajavel Elango, Carla M. Prado

**Affiliations:** 1 Human Nutrition Research Unit, Department of Agricultural, Food & Nutritional Science, University of Alberta, Edmonton, AB T6G 2R3, Canada; apagano@ualberta.ca (A.P.P.); kford@ualberta.ca (K.L.F.); 2 Women and Children’s Health Research Institute, Edmonton, AB T6G 1C9, Canada; 3 Division of Geriatrics, Department of Medicine, Duke University School of Medicine, Durham, NC 27705, USA; kathryn.starr@duke.edu; 4 Durham VA Health Care System, Durham, NC 27705, USA; 5 Institute for Physical Activity and Nutrition, Deakin University, Geelong 3217, Australia; nicole.kiss@deakin.edu.au; 6 Department of Obstetrics and Gynecology, University of Alberta, Edmonton, AB T6G 2R3, Canada; helen.steed@albertahealthservices.ca; 7 John W. Scott Health Sciences Library, University of Alberta, Edmonton, AB T6G 2R7, Canada; janice.kung@ualberta.ca; 8 Department of Pediatrics, BC Children’s Hospital Research Institute, School of Population and Public Health, University of British Columbia, Vancouver, BC V5Z 4H4, Canada; relango@bcchr.ubc.ca

**Keywords:** energy metabolism, energy expenditure, resting energy expenditure, energy needs, nutrition assessment, cancer, gynecological cancers, review

## Abstract

Determining energy requirements is vital for optimizing nutrition interventions in pro-catabolic conditions such as cancer. Gynecological cancer encompasses the most common malignancies in women, yet there is a paucity of research on its metabolic implications. The aim of this review was to explore the literature related to energy metabolism in gynecological cancers. We were particularly interested in exploring the prevalence of energy metabolism abnormalities, methodological approaches used to assess energy metabolism, and clinical implications of inaccurately estimating energy needs. A search strategy was conducted from inception to 27 July 2021. Studies investigating energy metabolism using accurate techniques in adults with any stage of gynecological cancer and the type of treatment were considered. Of the 874 articles screened for eligibility, five studies were included. The definition of energy metabolism abnormalities varied among studies. Considering this limitation, four of the five studies reported hypermetabolism. One of these studies found that hypermetabolism was more prevalent in ovarian compared to cervical cancer. Of the included studies, one reported normometabolism at the group level; individual-level values were not reported. One of the studies reported hypermetabolism pre- and post-treatment, but normometabolism when re-assessed two years post-treatment. No studies explored clinical implications of inaccurately estimating energy needs. Overall, commonly used equations may not accurately predict energy expenditure in gynecological cancers, which can profoundly impact nutritional assessment and intervention.

## 1. Introduction

**Figure 1:**
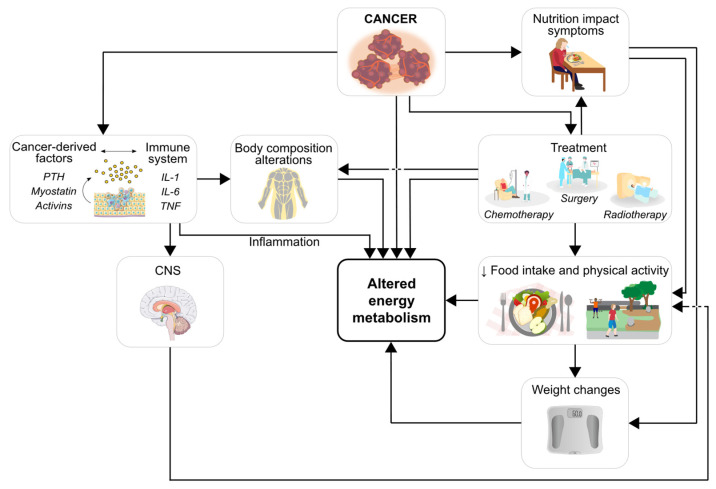
Cancer (including gynecological cancers), with or without the presence of metastasis, may lead to altered energy metabolism. Arrows indicate select pathways that contribute to this process. Cancer stimulates the secretion of cancer-derived factors (e.g., PTH, myostatin, and activins) that interact with the immune system, promoting the secretion of pro-inflammatory cytokines (e.g., IL-1, IL-6, and TNF). The resulting inflammatory state may directly or indirectly impact energy metabolism. Indirect changes happen through alterations in body composition and/or in the balance of orexigenic and anorexigenic neuropeptides in the CNS. Changes in this balance may lead to decreased food intake and levels of physical activity. Cancer and/or its treatment may induce nutrition impact symptoms (e.g., nausea, loss of appetite, constipation, diarrhea) that can also lead to reduced food intake and physical activity patterns, which in turn can promote weight changes. In summary, cancer, its treatment (and associated side-effects), inflammatory status, body composition alterations, reduced food intake and physical activity, and weight changes may lead to altered energy metabolism in cancer. PTH, parathyroid hormone; IL-1, interleukin-1; IL-6, interleukin-6; TNF, tumor necrosis factor; CNS, central nervous system. Images retrieved from smart.servier.com (accessed on 5 May 2022).

Gynecological cancers affect millions of women worldwide. The five most common types include: cervix, ovaries, uterus, vagina, and vulva. In 2020, ovarian, cervical, and uterine cancers were among the top ten most common cancers diagnosed in women worldwide, and cervical cancer had the third highest age-standardized rate for cancer incidence and mortality among them [1]. Side-effects from the disease and/or treatment can alter nutritional status and consequently nutritional needs in patients with cancer, including women with gynecological malignancies, as shown in Figure 1. Altered energy needs may contribute to the development of malnutrition [2,3], a condition associated with low muscle mass [4]. Both malnutrition and low muscle mass are frequently observed in any cancer stage and in patients with any body mass index (BMI) [5,6]. An appropriate supply of energy is therefore essential to combat the energetic demand of the tumor and prevent unintentional weight and muscle losses [5,7], but also to avoid increases in fat mass in the context of overweight and obesity. Excess body weight is commonly observed in this population, especially in those with endometrial cancer [8]. Thus, the accurate quantification of energy needs is warranted to promote optimal energy recommendations during treatment for gynecological cancers.

In clinical practice, energy requirements are estimated from resting energy expenditure (REE), the largest component of total energy expenditure (TEE) [9]. When available, indirect calorimetry should be used to assess REE in patients suspected to have altered energy metabolism or when conventional nutritional support fails to respond to the patients’ needs [10]. Cost and accessibility are among factors that limit the use of this technique in clinical settings [11], suggesting that less costly portable devices are needed. Predictive equations (e.g., Harris–Benedict equation or an estimation based on kilocalories per kilogram of body weight) have been used and/or recommended as a viable alternative to estimate energy needs due to their practicality and low cost [12,13].

Despite the incidence of gynecological cancers and the potential effect of the malignancy and its treatment on energy needs, there is a paucity of research on the metabolic demands in these types of cancer. Thus, the objective of this scoping review was to explore the literature related to energy metabolism in gynecological cancers. We were particularly interested in exploring the prevalence of energy metabolism abnormalities, methodological approaches used to assess energy metabolism, and clinical implications of inaccurately estimating energy needs.

## 2. Materials and Methods

This scoping review is reported according to the Preferred Reporting Items for Systematic Reviews and Meta-Analyses extension for Scoping Reviews (PRISMA-ScR) [14]. A protocol was developed but not registered. The medical librarian developed and executed a comprehensive search strategy in Ovid MEDLINE, Ovid Embase, CINAHL, and ProQuest Dissertations & Theses Global, from inception to 27 July 2021. All relevant keywords and controlled vocabulary pertaining to energy metabolism and gynecological cancers were included in the search strategies, which were limited to the English language. The full-text search strategy is included as Supplementary Material.

**Figure 2:**
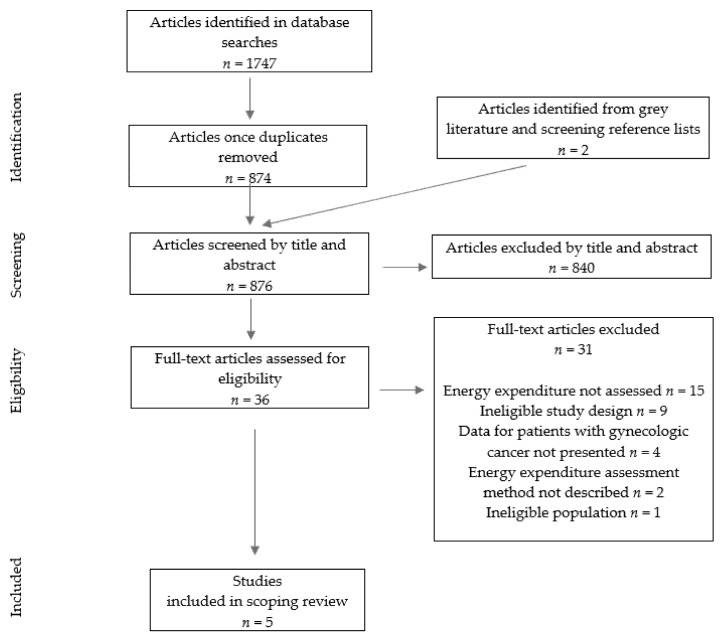
Flow diagram describing the inclusion process of studies investigating energy metabolism in gynecological cancer.

Search results were imported into covidence.org (Covidence, Melbourne, Australia) for screening. After duplicates were removed, titles and abstracts were screened for eligibility. In addition to subscription databases, the first 200 Google Scholar results and bibliographies from included studies were screened for pertinent articles. A Preferred Reporting Items for Systematic Reviews and Meta-Analyses (PRISMA) flow chart illustrating the selection process of included articles is shown in Figure 2 [14]. Two independent reviewers screened articles of interest according to title and abstract and subsequently completed a full-text review of relevant studies. In the case of disagreement, a third reviewer completed the article screening process.

Eligibility criteria included observational studies and randomized controlled trials (RCTs) with baseline data on energy metabolism of adult females (≥18 years, neither pregnant nor breastfeeding) with any stage or type of gynecological cancer, regardless of type of cancer treatment or route of nutrition delivery. Studies that measured energy metabolism using an accurate technique (e.g., indirect calorimetry) and incorporated a combined analysis for one or more types of gynecological cancers were included. Studies that assessed energy metabolism without reporting its specific findings, or combined analyses of different cancers other than gynecological cancer, without providing the gynecological data separately, were excluded. Previously published reviews, meta-analyses, and case-reports were also excluded.

Abnormality in energy metabolism was defined as the difference between measured and predicted REE within each study. As approximately 85% of the healthy population has a measured REE between 90 and 110% of that predicted by equations, this range was used to define altered metabolism [15]. If a range was not provided by the included study, calculations were made using available data. Predicted REE was determined using the Harris–Benedict equation for females [16]. When a study reported energy metabolism using a metric that could not be converted to kilocalories for comparison against predictive equations, the presence of energy metabolism abnormalities was assessed based on reported data.

## 3. Results

As shown in Figure 2, the initial search yielded 1747 articles of which five were eligible and included in this scoping review. Selected articles were published between 1936 and 2012 and are summarized in Table 1. Two of the included articles were cross-sectional studies [17,18], two reported on a longitudinal study [19,20], and one was an RCT [21].

The sample size of included studies, considering only gynecological cancers, varied from *n* = 15 to *n* = 124 and the mean age ranged from 49 to 60 years old, with the exception of de la Maza et al., who reported age as a range (27–60 years old) [20]. Of the included studies, participants were, on average, within a normal BMI range in the study by Dickerson et al. [18], in a normal to overweight BMI range (18.5–25 kg/m^2^) in the study by Macciò et al. [21], and classified as overweight (mean BMI before treatment: 27.3 ± 4.8 kg/m^2^; end of treatment: 27 ± 4.8 kg/m^2^; two years post-treatment: 28.5 ± 5.8 kg/m^2^) in the studies by de la Maza et al. [19,20]. Notably, different mean values of BMI were reported in the two studies by de la Maza et al.; however, the source of discrepancy was unclear [19,20]. Data on participants’ weight or BMI were not provided in the study by Bowman et al. [17].

The energy metabolism of women diagnosed with cervical (*n* = 30) and ovarian (*n* = 31) cancers was investigated at the individual level in the study by Dickerson et al. [18], while mixed gynecological cancers were explored in the other four studies. Bowman et al. studied women (*n* = 38) with ovarian (*n* = 2), cervical (*n* = 27), tubal (*n* = 1), uterine (*n* = 5), vaginal (*n* = 2), and vulvar (*n* = 1) cancers [17]. Macciò et al. included women (*n* = 124) with advanced-stage gynecological cancers: ovarian (*n* = 50), endometrial (*n* = 49), and cervical (*n* = 25) [21]. de la Maza et al. included women (*n* = 15) receiving radiotherapy for gynecological cancers: cervical (*n* = 10), endometrial (*n* = 3), uterine (*n* = 1), and vaginal (*n* = 1) [20]. The same authors performed a follow-up assessment 2 years post-cancer treatment with the same sample; results were presented in a different publication [19].

Four out of five studies provided information on cancer stage. From those, three studies included both early (stage I or II) and advanced stages (III or IV) [18,19,20], while one included only patients with advanced disease [21]. Sub-analysis considering cancer stage was not performed in the included studies with the exception of Dickerson et al. who stratified participants (*n* = 53) by early versus advanced stage, and found no difference in measured versus predicted REE [18]. In the study by Macciò et al., patients had received at least one line of chemotherapy treatment and several were receiving weekly low-dose regimens of concomitant palliative chemotherapy at the time of assessments [21]. In the studies by de la Maza et al., 15 patients had received five weeks of pelvic external radiotherapy [19,20]. Of those, 14 patients had additionally received intracavitary radiotherapy after external radiation, and seven had undergone surgery (hysterectomy plus oophorectomy) prior to radiation [19,20]. Cancer treatment regimens were not reported in the studies by Dickerson et al. and Bowman et al. [17,18].

The nutrient intake of patients included in the studies varied. Dickerson et al. studied patients receiving parenteral or enteral nutrition support with minimal (<500 kcal/day) or no ad libitum oral intake at the time of measurements [18]. Based on the nature of the assessments performed in the studies by de la Maza et al., Macciò et al., and Bowman et al., we inferred oral intake; however, we cannot determine if an alternative feeding route was also used [17,19,20,21].

Body composition was assessed by dual-energy X-ray absorptiometry (DXA) in the studies by de la Maza et al. and Macciò et al. using a Lunar DPX-L densitometer (LUNAR Corp. Madison, WI, USA) [19,21] and a Hologic Delphi W scanner (Hologic Inc., Bedford, MA, UAS), respectively [18]. When comparing pre- and end of treatment changes to body composition, de la Maza et al. observed a reduction in fat-free mass (kg; *p* = 0.005) but no changes to fat mass [20]. Weight loss was also observed (*p* = 0.03) and primarily constituted by a loss of fat-free mass given that there was no difference observed in percent fat-free mass [20]. When comparing data from pre- and end of treatment to two years post-treatment, weight (*p* < 0.01) and fat mass (percent and kg; both *p* < 0.01) increased while percent fat-free mass decreased (*p* < 0.01) [19]. Notably, the pre- and end-of-treatment body composition data reported in the two studies by de la Maza et al. did not match, and the source of discrepancy remained unclear [19,20]. In the study by Macciò et al., the lean mass (kg) of patients with gynecological cancer was provided, although an analysis to explore differences between study arms at baseline was not performed [21]. Data were not standardized by height; thus, we cannot further assess body composition in this group. Of note, we are unable to confirm exactly which body composition compartment was being measured, as the equation used to predict fat-free mass or lean mass in these studies was not provided.

Resting energy expenditure was measured in four [18,19,20,21] of the five studies, while basal metabolic rate (BMR) was measured in one study [17]. In the study by Dickerson et al., the REE of inpatients was measured at different times of the day following a minimum two-hour fast to reduce the effect of diet-induced thermogenesis [18]. The three other studies did not specify their energy metabolism assessment protocol [19,20,21], although one reported following “standard techniques” [19]. In the one study measuring BMR, Bowman et al. reported that inpatients remained in bed and were wheeled to their BMR assessment after a 16-h fast [17]. The average of two BMR assessments was reported [17]. Notably, except for the studies by de la Maza et al. [19,20], none of the studies herein provided information on participants’ menstrual cycle. In the longitudinal study by de la Maza et al., seven participants were menopausal prior to cancer treatment, while the rest (*n* = 8) became menopausal post-treatment [19].

Indirect calorimetry was used to measure energy expenditure in all studies included herein although devices varied. The Medgem^®^ (SensorMedics Italia Srl, Milan, Italy) was used by Macciò et al. [21]; the Sensor Medic model 2900 calorimeter (further device specifications not available) was used in both studies by de la Maza et al. [19,20]; the Benedict–Roth apparatus was used by Bowman et al. [17]; and equipment specifications were not provided by Dickerson et al. [18]. Total energy expenditure was estimated in one [19] of the five studies reviewed, although the method used to obtain TEE (REE multiplied by the activity energy expenditure from patient-reported daily physical activity) may not be accurate [22] and thus is not discussed herein.

In the study by Dickerson et al., the mean measured REE of patients with ovarian cancer (1332 ± 214 kcal/day) was higher than those with cervical cancer (1179 ± 181 kcal/day; *p* < 0.01) [18]. Notably, mean age and body weight did not differ between cancer types within this study [18]. Similarly, patients with ovarian cancer had a significantly higher measured versus predicted REE (109 kcal ± 18% of predicted REE) compared to patients with cancer of the cervix (98 kcal ± 16% of predicted REE; *p* < 0.02). When comparing the measured REE with the REE predicted by the Harris–Benedict equation, the measured REE was higher (1234 ± 115 kcal/day) in the ovarian cancer group (*p* < 0.02), but no difference was found in the cervical cancer group [18]. Hypermetabolism was more prevalent (55%) in the ovarian cancer group in relation to the cervical cancer group (13%) [18]. Overall, 45% of patients, regardless of cancer type, had a measured REE within ±10% of that predicted by equations, 21% were hypometabolic, and 34% were hypermetabolic [18].

In the study by Macciò et al., the mean measured REE of patients with gynecological cancer at baseline did not seem to differ between study arms prior to intervention; however, an analysis to determine statistical significance was not presented by the authors [21]. The mean measured REE was within 99% of the group-level mean REE predicted by the Harris–Benedict equation, suggesting normometabolism, on average, in this sample [21]. We were unable to determine the prevalence of abnormalities at the individual level in this population.

In the studies by de la Maza et al., the REEs measured prior to cancer treatment and post-treatment were higher when compared to that measured two years post-treatment (*p* < 0.01) [19,20]. Compared to the REE predicted by the Harris–Benedict equation, the measured REE was 1.24 ± 0.2 times higher prior to treatment, 1.21 ± 0.16 times higher after treatment, and 0.92 ± 0.06 times higher two years post-treatment [19,20]. The authors did not compare or report the percentage of patients presenting with a measured REE within ± 10% of the predicted REE [19]. Furthermore, the individual-level predicted REE was not provided in kcal/day; thus, we are unable to determine the prevalence of hypo-, hyper-, or normo-metabolism in this study [19]. However, the mean measured REE of the study sample was within 124%, 121%, and 92% of the mean REE predicted by the Harris–Benedict equation during pre-treatment, at completion of treatment, and two years post-treatment, respectively [19].

Energy expenditure was expressed as the percentage of deviation from normal in the study by Bowman et al., although the definition of “normal” was not provided [17]. Results from this study indicated that energy expenditure in gynecological cancer had a minimum and a maximum deviation of −13.6% and +53.5% from normal, respectively (mean: +9.14%) [17]. The mean percent deviation from normal appeared higher in women with cancer compared to the non-cancer control group (minimum: −12.7%, maximum: +30%, mean: +2.5%) [17].

Overall, the mean measured REE could be compared with the REE predicted by equations in four of the five studies included herein [18,19,20,21], while the deviation from normal was used in one study [17]. Hypermetabolism was identified in four [17,18,19,20] of the five studies included herein, with one further indicating normometabolism two years after cancer treatment completion [19]. Although Dickerson et al. did not find abnormalities in mean REE at a group level for patients with cervical cancer, abnormalities were identified at the individual level for their entire sample (including ovarian cancer) with the measured REE ranging from 53 to 157% of the predicted REE [18]. Among the five studies included herein, none explored the clinical implications of inaccurately estimating energy needs in this cancer cohort.

## 4. Energy Metabolism in Gynecological Cancers: Current Knowledge

To our knowledge, this is the first scoping review on energy metabolism characteristics in gynecological cancers. Data from studies included herein suggest that patients with gynecological cancer presented with abnormalities in energy metabolism, and that predictive equations may be inaccurate to detect true metabolic demands in this population. These findings are in line with the literature whereby energy metabolism abnormalities vary greatly in cancer, therefore altering energy requirements [23]. In the context of cancer, disease-related factors including tumor burden, location, stage, type of treatment, and the presence of metastasis can lead to derangements in energy metabolism, more specifically, in REE [2].

Accurate assessment of energy metabolism is vital for optimizing nutritional interventions, avoiding malnutrition, and addressing the challenge of variable energy expenditure in cancer. Compared with those without a malignancy, women with cancer had a higher REE [17]. Similarly, the REE of survivors (two years post-cancer treatment) decreased compared to pre- and end of treatment [19]. In this longitudinal study, de la Maza et al. suggested that the two-year post-treatment reduction in REE was a correction to a normometabolic state following a moderately hypermetabolic phase pre-treatment [19]. Interestingly, and in line with the literature in other cancer types, not all studies reported alterations in energy metabolism [21]. Given the highly variable energy metabolism profiles in cancer [2], current oncology nutrition guidelines for energy intake are stipulated based on healthy populations [13].

Energy expenditure also varied across cancer subtypes; for instance, a higher REE was observed in women with ovarian cancer when compared with those with cervical cancer [18]. Notably, screening for cervical cancer seems to be much more specific than the screening for ovarian cancer, resulting in ovarian cancer often being diagnosed at later stages, which may affect energy metabolism [24]. Additionally, ovarian cancer metastases (i.e., advanced disease) primarily affect the gastrointestinal tract and can result in significant nutrition impact symptoms and poor nutritional status [25]. Differences in REE could have been influenced by the higher prevalence of advanced disease (i.e., stages III–IV) among patients with ovarian cancer, but findings were not statistically significant in a sub-analysis [18]. However, it is possible that the study was underpowered for this analysis (*n* = 27 ovarian versus *n* = 10 cervical)) [18].

Notably, REE was measured in four studies [18,19,20,21] and BMR in one [17]. Although often used interchangeably, REE and BMR differ. Resting energy expenditure is higher than BMR as it is measured at any time of the day, after 10 to 20 min of rest [26,27,28], while BMR is assessed after waking from sleep; thus, it is assessed in the morning, while the individual is at complete rest [29].

Energy expenditure is influenced by several factors including body composition, which was assessed in three of the five studies included herein [19,20,21]. In the study by de la Maza et al., a ratio of fat-free mass to REE was reported [19]. This method of data presentation introduces a bias related to body size as fat-free mass is likely to be higher in those living in larger body sizes, with the exception of sarcopenic obesity (i.e., concurrent presentation of low muscle mass and elevated adiposity) [30]. Additionally, fat-free mass is composed of organs and tissues with varying metabolic activity levels, which will impact REE differently across individuals regardless of increased fat-free mass [30]. Menstrual cycle and menopause are additional factors that influence energy expenditure. Pre-menopausal women have a higher energy expenditure compared to those who are post-menopausal [31]. Additionally, differences in energy expenditure are observed during the menstrual cycle. Higher progesterone levels across the menstrual cycle in the postovulatory phase may increase REE [31,32]. In a study of 10 menstruating women, energy expenditure increased 9% (range 8–16%) during the fourteen- day luteal phase following ovulation [32]. Changes in hormone levels during menopause may also explain differences in energy in these women [31]. Thus, the phase of menstrual cycle and/or presence of menopause should be considered when interpreting findings.

Abnormalities in energy metabolism can be determined by comparing measured to predicted (i.e., by equations) REE. However, results may vary according to the technique and protocol used to measure REE and by the equation chosen to predict REE. For instance, the Harris–Benedict equation is one of the most used predictive equations [33], but it was developed using a healthy population and in 1918 when people were leaner compared to contemporary populations [15]. The Harris–Benedict equation may lead to the erroneous estimation of energy requirements regardless of body weight, but particularly by overestimating REE in individuals with normal weight or obesity [33]. Errors may be even more substantial if equations are used with individuals who have specific health conditions or characteristics (such as those with cancer), which were not included in the analysis [12]. However, overestimation was not reflected in the findings from the study by Macciò et al., where participants were normometabolic despite having a normal weight [21] and in the study by de la Maza et al. where participants were normometabolic two years post-cancer treatment despite having excess body weight (i.e., overweight at the group level) [19]. Many additional predictive equations exist and can include different parameters (e.g., age, sex, fat-free mass, fat mass). The addition of these variables may increase population-specific precision, especially given the numerous factors that affect energy metabolism. The variability in energy metabolism reported in the studies herein may reflect the poor accuracy of predictive equations for this patient population. In fact, the reduced accuracy of predictive equations has been demonstrated in other cancer types [12]. For example, a study comparing the accuracy of measured REE to 23 predictive equations in a mixed cancer cohort found that the equations were inaccurate on an individual level [12]. Despite the practicality of using predictive equations, determining accurate energy requirements is essential to meet patients’ true metabolic demands, ultimately optimizing body composition and nutritional status [4].

Based on the findings of the studies reported herein, we cannot make any assumptions or generate any hypotheses regarding the implications of inaccurately estimating energy needs in gynecological malignancies. However, adequate estimation of the energy needs is essential for providing accurate energy recommendations. An adequate energy intake is essential for skeletal muscle health and is associated with patient outcomes [34]. Low muscle mass in cancer has been associated with physical disability, extended hospitalization, infectious and noninfectious complications, increased risk for severe chemotherapy toxicity, and shorter survival [5,34,35,36,37]. Thus, future studies should investigate associations between estimated and actual energy needs and the corresponding health implications in individuals with gynecological cancers. Such information would provide further insight into the indispensable role of nutrition for cancer care and would be valuable information for guideline creation and implementation. Such an understanding is a key step in advocating for nutritional assessment and intervention upon cancer diagnosis, regardless of disease stage or BMI. Early and targeted nutritional interventions may improve patients’ nutritional status during and beyond cancer, ultimately improving patients’ prognosis.

Notably, these findings on energy metabolism abnormalities in gynecological cancers are hypothesis-generating as they are derived from a very limited number of studies, despite the unlimited publication date applied in our search criteria. Inconsistencies in study design, data analyses, and presentation of findings make it difficult to characterize the energy metabolism profiles and abnormalities in women with this cancer type. Importantly, the equipment used to measure REE in all included studies only measured oxygen consumption but not carbon dioxide production, which may be a less accurate (or inaccurate) method of REE assessment. For instance, a different indirect calorimetry device (FitMate GS) that solely measures oxygen consumption was compared to the metabolic cart in people with cancer and found poor accuracy at the individual level despite minimal errors when group means were compared [38]. Additionally, the MedGem device was found to be inaccurate at the individual level in a different cohort of patients (adults living with class II or III obesity) [39]. This device has also been found to underestimate REE, although being reliable for repeated measurements [11]. Lastly, menstrual cycle information should be considered when measuring energy needs of women because energy expenditure can increase significantly depending on the phase of the cycle [32].

## 5. Future Directions

Future studies should use accurate techniques to explore the energy metabolism profile of women with gynecological cancer. For REE, this includes indirect calorimeters that measure both oxygen and carbon dioxide. Further, improving the accuracy of predictive equations is particularly relevant in the context of clinical settings, as the ones currently available do not appear adequate for this population. There is also a need to understand if and how longitudinal changes in energy metabolism occur, and to seek to determine the factors associated with these potential changes (e.g., inflammatory status, body composition, cancer treatment). Importantly, the majority of the studies included herein were published more than twenty years ago, with the most recent article published ten years ago. Cancer treatments have evolved over the past decade; thus, future studies should investigate the energy needs of patients with gynecological cancer in the context of current treatments. These findings may highlight the need to adjust energy intake recommendations based on cancer type and/or stage and have the potential to help identify drivers of energy metabolism abnormalities during and beyond cancer. Future studies should consider sample characteristics that impact energy needs such as physical activity patterns, BMI or body weight changes, and phases of menstrual cycle. Future research in the field may be facilitated with the development of methodological standards/guidelines endorsed by relevant societies. Such endeavors would promote the standardization and/or best practices for trials focusing on energy expenditure in cancer.

## 6. Conclusions

This scoping review revealed the scarcity of research investigating energy metabolism in patients diagnosed with gynecological cancer. Findings on energy metabolism profiles in gynecological cancers are hypothesis-generating and suggest that women with these malignancies are likely to present with energy metabolism abnormalities (i.e., hypermetabolism). It is possible that metabolic demands may be different across different gynecological cancer types (e.g., cervical versus ovarian), as well as throughout the cancer trajectory (e.g., pre- versus post-treatment). Lastly, predictive equations may not accurately represent true metabolic demands in this cancer type.

Our results must be interpreted in view of the limited number of articles, the small sample size, inconsistencies in study design (e.g., timepoints, data analyses, presentation of findings), and limitations of the type of equipment used to assess energy metabolism reported herein. As such, no substantial conclusions can be drawn in regard to the prevalence of energy metabolism abnormalities in people with gynecological cancers.

Overall, this scoping review highlights the need for further research on energy metabolism in gynecological cancer, which may help clarify whether specific cancer types are associated with different energy needs. Providing targeted, evidence-based energy recommendations to patients can optimize nutritional status, improving the response to cancer treatment and survivorship.

## Figures and Tables

**Table 1:** Summary of five studies investigating energy metabolism in gynecological cancer.

Author, Year	Mean Age (Years)	Cancer Type (*n*)	Cancer Stage ^1^	Cancer Treatment	Energy Expenditure (kcal/Day) Measured Predicted		Abnormalities in Energy Metabolism	Technique/Equation
Bowman et al., 1936 [17]	54	Ovarian (*n* = 2); Cervical (*n* = 27); Tubal (*n* = 1); Uterine (*n* = 5); Vaginal (*n* = 2); Vulvar (*n* = 1)	Not reported	Not reported	Not reported	Not reported	Hypermetabolism ^2^ Deviation from “normal ^3^” ranged from −13.6% to +53.5%; average deviation: +9.14%	Benedict–Roth apparatus/not specified
Dickerson et al., 1995 [18]	Cervical: 55 Ovarian: 58	Cervical (*n* = 30); Ovarian (*n* = 31)	I-II: Cervical (*n* = 15); Ovarian (*n* = 1) III-IV: Cervical (*n* = 10); Ovarian (*n* = 27) Unknown: Cervical (*n* = 5); Ovarian (*n* = 3)	Not reported	Ovarian: 1332 ± 214 Cervical: 1179 ± 181	Ovarian: 1234 ± 115 Cervical: 1203 ± 118	Hypometabolic: 21% Hypermetabolic: 34% Normometabolic: 45%	Indirect calorimetry (device not specified)/Harris–Benedict
de la Maza et al., 2001 [20]	*n*/A ^4^	Cervical (*n* = 10); Endometrial (*n* = 3); Uterine (*n* = 1); Vaginal cupula (*n* = 1)	IB: Cervical (*n* = 5) IC: Endometrial (*n* = 2) II: Endometrial (*n* = 1) IIB: Cervical (*n* = 3) IIIB: Cervical (*n* = 2) Unknown: Uterine (*n* = 1); Vaginal cupula (*n* = 1)	45–50 Gy of pelvic external radiation over 5 weeks	Pre-treatment ^5^: 1673 ± 488 Post-treatment ^5^: 1585 ± 275	Not reported or possible to calculate	Pre-treatment: Hypermetabolism (measured REE 125% of predicted) Post-treatment: Not reported	Sensor Medic model 2900 calorimeter/Harris–Benedict
de la Maza et al., 2004 [19]	49	Cervical (*n* = 10); Endometrial (*n* = 3); Uterine (*n* = 1); Vaginal cupula (*n* = 1)	IB: Cervical (*n* = 5) IC: Endometrial (*n* = 2) II: Endometrial (*n* = 1) IIB: Cervical (*n* = 3) IIIB: Cervical (*n* = 2) Unknown: Uterine (*n* = 1); Vaginal cupula (*n* = 1)	45–50 Gy of pelvic external radiation over 5 weeks (*n* = 15) and post-external ratiation intracavitary radiotherapy (*n* = 14) and/or surgery prior to external radiation (*n* = 7)	Pre-treatment ^5,6^: 1690 ± 231 Post-treatment: 1644 ± 292 Two years post-treatment: 1287 ± 175	Pre-treatment ^5,6^: 1363 Post-treatment: 1359 Two years post-treatment: 1399	Hypermetabolism (pre- and post-treatment); normometabolism two years post-treatment	Sensor Medic model 2900 calorimeter/Harris–Benedict
Macciò et al., 2012 [21]	60	Ovarian (*n* = 50); Endometrial (*n* = 49); Cervical (*n* = 25)	IIIC: Ovarian (*n* = 5) IV: Ovarian (*n* = 45); Endometrial (*n* = 49); Cervical (*n* = 25)	Previous chemotherapy (*n* = 124) and/or ongoing palliative chemotherapy (*n* = 90)	Baseline (prior to intervention): Arm 1: 1166 ± 440 Arm 2: 1157 ± 279	Baseline (prior to intervention) ^7^: Arm 1: 1146 Arm 2: 1155	Normometabolism	Medgem^®^/Harris–Benedict

^1^ Studies by de la Maza et al. (2001 and 2004) [19,20] and Dickerson et al. (1995) [18] used the International Federation of Gynecology and Obstetrics (FIGO) staging classification system; other studies did not specify staging system used. ^2^ Abnormalities in energy metabolism were not classified based on measured REE within ±10% of that predicted by equations. ^3^ “Normal” was not defined. ^4^ Mean age was not available (*n*/A) in the study by de la Maza et al. (2001) [20]; a range of 27–60 years old was provided instead. ^5^ Pre-treatment and post-treatment values were reported by de la Maza et al. (2001) [20] and later presented in their 2004 study [19]. Values were presented in kcal/day in their 2001 study [20] and in kJ/day in the 2004 study [19]. Differences in values after conversion were observed for both mean REE and standard deviation values, and it is unclear where the discrepancy arose from. ^6^ Values originally reported as kJ per day. For consistency, kJ was converted to kcal. ^7^ Group-level mean values of age, height, and weight reported at baseline for each study arm were used to estimate group-level predicted REE using the Harris–Benedict equation for females.

## Data Availability

Not applicable.

## Supplementary Materials

The following supporting information can be downloaded at: https://www.mdpi.com/article/10.3390/ijerph19116419/s1, Table S1: Search strategies performed in Ovid MEDLINE, Ovid Embase, CINAHL, ProQuest Dissertations & Theses Global, and Google Scholar.
