# Supplementary material for: Energy Metabolism in Gynecological Cancers: A Scoping Review
Source: Int J Environ Res Public Health. 2022 May 25;19(11):6419. doi: 10.3390/ijerph19116419 (PMC9180127; doi:10.3390/ijerph19116419)
Supplement: Supplementary file 1 [file ijerph-19-06419-s001.zip › ijerph-1687304-supplementary.pdf]

**Table S1:** Search strategies performed in Ovid MEDLINE, Ovid Embase, CINAHL, and ProQuest Dissertations & Theses Global, and Google Scholar

| **Database** | **Search Strategy** |
| --- | --- |
| MEDLINE<br><br>Ovid MEDLINE(R) ALL 1946 to July 26, 2021 | 1. ((energy or caloric) adj3 (needs or intake)).mp.<br>2. energy expenditure*.mp.<br>3. energy metabolism*.mp.<br>4. caloric expenditure*.mp.<br>5. resting metabolic rate.mp.<br>6. basal metabolic rate.mp.<br>7. basal metabolism.mp.<br>8. indirect calorimetr*.mp.<br>9. respiration calorimetr*.mp.<br>10. or/1-9<br>11. exp Ovarian Neoplasms/<br>12. exp Endometrial Neoplasms/<br>13. exp Uterine Cervical Neoplasms/<br>14. ((ovarian or ovary or endometri* or cervix or cervical or gyn?ecologic* or uterus or uterine) adj3 (cancer* or neoplasm* or carcinoma* or oncolog* or malignan* or tumo?r* or metasta*)).mp.<br>15. or/11-14<br>16. 10 and 15<br>17. limit 16 to english language<br>18. animals/<br>19. humans/<br>20. 18 not (18 and 19)<br>21. (veterinary or rabbit or rabbits or animal or animals or mouse or mice or rodent or rodents or rat or rats or murine or hamster* or pig or pigs or piglets or swine or porcine or horse* or equine or cow or cows or cattle or bovine or goat or goats or sheep or lambs or ovine or monkey or monkeys or trout or marmoset$1 or canine or dog or dogs or |

| | |
|---|---|
| | feline or cat or cats or zebrafish).ti.<br>22. 20 or 21<br>23. 17 not 22 |
| Embase<br><br>Ovid Embase 1974 to 2021 July 26 | 1. ((energy or caloric) adj3 (needs or intake)).mp.<br>2. energy expenditure*.mp.<br>3. energy metabolism*.mp.<br>4. caloric expenditure*.mp.<br>5. resting metabolic rate.mp.<br>6. basal metabolic rate.mp.<br>7. basal metabolism.mp.<br>8. indirect calorimetr*.mp.<br>9. respiration calorimetr*.mp.<br>10. or/1-9<br>11. exp ovary tumor/<br>12. exp endometrium tumor/<br>13. exp uterine cervix tumor/<br>14. ((ovarian or ovary or endometri* or cervix or cervical or gyn?ecologic* or uterus or uterine) adj3 (cancer* or neoplasm* or carcinoma* or oncolog* or malignan* or tumo?r* or metasta*)).mp.<br>15. or/11-14<br>16. 10 and 15<br>17. limit 16 to english language<br>18. animal/<br>19. human/<br>20. 18 not (18 and 19)<br>21. (veterinary or rabbit or rabbits or animal or animals or mouse or mice or rodent or rodents or rat or rats or murine or hamster* or pig or pigs or piglets or swine or porcine or horse* or equine or cow or cows or cattle or bovine or goat or goats or sheep or lambs or ovine or monkey or monkeys or trout or marmoset$1 or canine or dog or dogs or |

| | |
|---|---|
| | feline or cat or cats or zebrafish).ti.<br>22. 20 or 21<br>23. 17 not 22 |
| CINAHL | S1 (energy or caloric) N3 (needs or intake)<br>S2 "energy expenditure*"<br>S3 "energy metabolism*"<br>S4 "caloric expenditure*"<br>S5 "resting metabolic rate"<br>S6 "basal metabolic rate"<br>S7 "basal metabolism"<br>S8 "indirect calorimetr*"<br>S9 "respiration calorimetr*"<br>S10 S1 OR S2 OR S3 OR S4 OR S5 OR S6 OR S7 OR S8 OR S9<br>S11 (MH "Ovarian Neoplasms+")<br>S12 (MH "Endometrial Neoplasms")<br>S13 (MH "Cervix Neoplasms+")<br>S14 (ovarian or ovary or endometri* or cervix or cervical or gyn#ecologic* or uterus or uterine) N3 (cancer* or neoplasm* or carcinoma* or oncolog* or malignan* or tumo#r* or metasta*)<br>S15 S11 OR S12 OR S13 OR S14<br>S16 S10 AND S15<br>S17 (MH "Animals+")<br>S18 (MH "Human")<br>S19 S17 NOT (S17 AND S18)<br>S20 TI veterinary or rabbit or rabbits or animal or animals or mouse or mice or rodent or rodents or rat or rats or murine or hamster* or pig or pigs or piglets or swine or porcine or horse* or equine or cow or cows or cattle or bovine or goat or goats or sheep or lambs or ovine or monkey or monkeys or trout or marmoset or marmosets or canine or dog or dogs or feline or cat or cats or zebrafish |

| | |
|---|---|
| | S21 S19 OR S20<br>S22 S16 NOT S21<br>S23 S16 NOT S21 (Limiter: English language) |
| ProQuest Dissertations & Theses Global | noft( (energy OR caloric) NEAR/3 (needs OR intake) OR ("energy expenditure" OR "energy expenditures") OR ("energy metabolism") OR "caloric expenditure*" OR "resting metabolic rate" OR "basal metabolic rate" OR "basal metabolism" OR ("indirect calorimetry") OR "respiration calorimetr*" ) AND ((noft(ovarian) or noft(ovary) or noft(endometri*) or noft(cervix) or noft(cervical) or noft(gyn?ecologic*) or noft(uterus) or noft(uterine)) NEAR/3 (noft(cancer*) or noft(neoplasm*) or noft(carcinoma*) or noft(oncolog*) or noft(malignan*) or noft(tumo?r*) or noft(metasta*))) NOT ti(veterinary or rabbit or rabbits or animal or animals or mouse or mice or rodent or rodents or rat or rats or murine or hamster* or pig or pigs or piglets or swine or porcine or horse* or equine or cow or cows or cattle or bovine or goat or goats or sheep or lambs or ovine or monkey or monkeys or trout or marmoset or marmosets or canine or dog or dogs or feline or cat or cats or zebrafish) |
| Google Scholar | ("energy expenditure" OR "basal metabolic rate" OR "indirect calorimetry") AND (ovarian cancer OR endometrial neoplasm OR cervical cancer OR gynecological cancer) |
